# Validation of the German Version of the Creative Imagination Scale (CIS) in Individuals with Mild to Moderate Depressive Symptoms: A Comparison with the HGSHS-5:G

**DOI:** 10.3390/bs16091502

**Published:** 2026-08-27

**Authors:** Julia Siewert, Benno Brinkhaus, Björn Rasch, Michael Teut, Maximilian Hinse

**Affiliations:** 1 Institute of Social Medicine, Epidemiology and Health Economics, Charité—University Medical Center Berlin, Corporate Member of Freie Universität Berlin and Humboldt-Universität zu Berlin, Charitéplatz 1, 10117 Berlin, Germany; benno.brinkhaus@charite.de (B.B.); mteut@gmx.de (M.T.); maximilian.hinse@charite.de (M.H.); 2 Department of Psychology, University of Fribourg, Rue P.A. de Faucigny 2, 1700 Fribourg, Switzerland; bjoern.rasch@unifr.ch; 3 Campus Brandenburg an der Havel, Brandenburg Medical School Theodor Fontane (MHB), Nikolaiplatz 19, 14770 Brandenburg an der Havel, Germany

**Keywords:** creative imagination scale, hypnotic suggestibility, validation study, psychometrics, factor analysis, depressive symptoms, Harvard Group Scale of Hypnotic Susceptibility

## Abstract

Imaginative suggestibility is an important component of hypnotic responsiveness. However, a validated German version of the Creative Imagination Scale (CIS) is lacking. This study aimed to evaluate the psychometric properties and convergent validity of the German CIS against the short form of the Harvard Group Scale of Hypnotic Susceptibility (HGSHS-5:G) in individuals with mild to moderate depressive symptoms. In this post hoc validation study, the German CIS was evaluated using reliability analyses, exploratory and confirmatory factor analyses, and mixed polychoric–tetrachoric correlations with the HGSHS-5:G. Among 97 participants, the CIS showed good internal consistency (α = 0.85, ω = 0.87). A two-factor model showed better fit than a unidimensional model, although absolute model fit remained suboptimal and the factors were highly correlated (r = 0.80). CIS and HGSHS-5:G scores were moderately correlated (r = 0.52, *p* < 0.001), supporting convergent validity. This first validation of the German CIS demonstrated good reliability and preliminary evidence for a two-factor structure, warranting replication in larger samples. The CIS may complement behavioral measures of hypnotic responsiveness in research and clinical applications.

## 1. Introduction

Hypnotic responsiveness is commonly assessed using behavioral scales that rely on a formal hypnotic induction and index hypnotic susceptibility, defined as responsiveness to suggestions delivered following induction. In contrast, the Creative Imagination Scale (CIS) was developed to assess imaginative suggestibility without induction, focusing on individuals’ capacity for voluntary imaginative involvement in suggested experiences.

Rather than assessing hypnotic susceptibility in the induction-dependent sense, the CIS captures responsiveness to non-induction-based suggestions and thus reflects the broader construct of suggestibility (Wilson & Barber, 1978). The CIS consists of ten standardized experiential suggestions—such as arm heaviness, imagined anesthesia, gustatory imagery, time distortion, and age regression—which respondents are instructed to visualize or experience through imagination.

Empirical studies and normative applications have demonstrated acceptable psychometric properties for the CIS, supporting its use as a valid measure of imaginative responsiveness to suggestion that is conceptually related to—but distinct from—hypnotic susceptibility (Barber & Wilson, 1978; Wilson & Barber, 1978).

In a series of initial investigations, normative data for the CIS were established, and the scale was shown to possess satisfactory psychometric properties, including test–retest reliability, split-half reliability, and factorial validity, supporting its coherence as a measure of imaginative responsiveness to suggestion (Barber & Wilson, 1978; Wilson & Barber, 1978).

Previous work has raised the question of whether imaginative suggestibility, as assessed by the CIS, is fully unitary. Factor-analytic investigations have suggested that CIS items may differentially reflect imagery-related absorption and performance-related perceptual alterations. In particular, Hilgard et al. (1981) reported a two-factor structure comprising an Absorption/Imagination factor and a Hypnotic Performance factor, with CIS items loading on both dimensions. At the same time, earlier descriptive and psychometric work had often treated the CIS as a largely unidimensional measure (e.g., Wilson & Barber, 1978).

Comparative research has further examined the construct validity of the CIS by comparing it with induction-based measures of hypnotic susceptibility. For example, McConkey et al. (1979) reported a modest positive correlation between CIS scores and scores on the Harvard Group Scale of Hypnotic Susceptibility, Form A (HGSHS:A; r = 0.28). Their findings indicate that, although the two measures share some variance related to suggestibility, they reflect largely distinct underlying dimensions, with the CIS primarily assessing imagery- and imagination-based processes.

Additional empirical work has reported acceptable internal consistency of the CIS and significant correlations with theoretically related constructs such as absorption, providing support for its reliability and convergent validity (Sapp & Hitchcock, 2003). In their study, the CIS demonstrated a coefficient alpha of approximately 0.84 and showed significant correlations with scores on the Tellegen Absorption Scale (TAS).

Although classical studies have reported modest positive correlations between the CIS and the full HGSHS:A (e.g., McConkey et al., 1979), direct empirical correlations between the CIS and the recently developed short form HGSHS-5:G (Harvard Group Scale of Hypnotic Susceptibility—5 item short form, German version) have not yet been reported.

Riegel and colleagues proposed a 5-item short version of the HGSHS:A to reduce test time and facilitate broader use. In German samples, the HGSHS-5:G demonstrated acceptable validity, reliability, and classification agreement relative to the original HGSHS:A (Riegel et al., 2021).

This reveals a research gap: although the HGSHS-5:G has been standardized in German as an economical measure of the motor challenge dimension of hypnotic susceptibility, a validated German version of the CIS is still lacking. Comparing both instruments in a clinical sample, such as individuals with mild to moderate depressive symptoms, may therefore provide important insights into their convergent and discriminant validity.

Although the CIS was originally developed to assess imaginative suggestibility in non-clinical contexts, examining the scale in individuals with depressive symptoms is theoretically meaningful because several CIS items rely on vivid imagination, absorption, and responsiveness to internally generated experiences. Mental imagery has been shown to evoke stronger emotional responses than verbal representation and may contribute to the maintenance of emotional disorders (Holmes & Mathews, 2010). Furthermore, in individuals with depressive disorders, negative mental imagery induced stronger negative affect compared to healthy controls, and a positive imagery deficit was observed in the explicit measure (Görgen et al., 2015). Given that depressive symptomatology is associated with imagery-related cognitive and affective processes, it may influence mechanisms relevant to responses on the CIS. Investigating CIS scores in individuals with mild to moderate depressive symptoms therefore provides an opportunity to examine the construct validity of the scale in a population in which mental imagery processes are relevant to depressive symptomatology. Importantly, the psychometric properties of suggestibility-related measures cannot be assumed to generalize from healthy to clinical populations, as depressive symptomatology is associated with alterations in cognitive, affective, and motivational processes that are directly relevant to imaginative engagement. Examining the CIS in a clinical sample thus allows for a more ecologically valid assessment of how imaginative suggestibility (CIS) and the motor challenge dimension of hypnotic susceptibility (HGSHS-5:G) differ in this population.

## 2. Materials and Methods

### 2.1. Participants and Procedure

Participants were recruited as part of the HypnoDeep randomized controlled trial, which examined the effects of telemedical hypnosis, progressive muscle relaxation, and routine care in individuals with mild to moderate depressive symptoms and elevated stress. Participants had clinically diagnosed depressive episodes, classified as mild according to ICD-10 criteria (International Classification of Diseases, 10th revision). Self-reported depressive symptoms at baseline, assessed with the Beck Depression Inventory–II (BDI-II), were in the mild to moderate range and were assessed as a continuous variable to characterize the sample. Perceived stress was assessed using the Perceived Stress Scale (PSS) and a visual analog scale for stress (VAS-Stress).

Recruitment was conducted in Germany via online advertisements, newsletters, and outpatient clinical networks. After an online screening for eligibility, participants were enrolled and provided written informed consent before participation.

In the original trial, participants were randomly assigned to one of 12 online groups: four received guided hypnotherapy, four progressive muscle relaxation, and four served as a wait-list control group without intervention. Questionnaires and demographic data were administered during the pre-intervention assessment phase. In total, 100 participants were enrolled and randomized in the HypnoDeep trial. Three participants were mistakenly randomized, did not complete any baseline assessments, and did not receive the allocated intervention. They were therefore excluded from all analyses. The present study is based on the remaining 97 participants, of whom 94 (97%) completed both the CIS and the HGSHS-5:G.

The final sample consisted of 69 women (71.1%), 26 men (26.8%), and 2 participants identifying as diverse (2.1%), with a mean age of 39.2 years (SD = 12.02; range = 20–68 years).

Inclusion criteria were: age between 18 and 70 years, mild to moderate depressive symptoms according to ICD-10 criteria (F32.0 or F33.0), and a self-reported stress level of ≥40 mm on a visual analog scale. Exclusion criteria included current or past psychotic disorder, post-traumatic stress disorder, personality disorder, acute suicidality, substance abuse, or severe medical illness that could interfere with participation.

### 2.2. Measures

#### 2.2.1. CIS

The CIS (Wilson & Barber, 1978) is a 10-item measure of imaginative suggestibility, designed to be administered without a formal hypnotic induction. Items tap into a range of imaginative experiences (arm heaviness, finger anesthesia, gustatory hallucinations, time distortion, age regression) and are rated on a 5-point scale according to participants’ subjective experience from 0 to 4 (0, not at all the same [experience]; 1, a little the same; 2, between a little and much the same; 3, much the same; 4, almost exactly the same), resulting in a total sum score between 0 and 40. A German CIS version based on the translation by Bahlinger (1995), which was used and documented in the diploma thesis by Egbers (1998; Imaginative Processing of Directly and Indirectly Formulated Texts Depending on Attentional Demand), was used in the present study. In the thesis, the full wording of the German CIS was provided in the appendix and the scale was used to assess imaginative ability. To our knowledge, however, no formal psychometric validation of this German version has previously been published. The translated items were administered in their original German wording as documented by Egbers (1998).

#### 2.2.2. HGSHS-5:G—5-Item Short Form

The HGSHS-5:G (Riegel et al., 2021; Zech et al., 2024) is a recently validated German short version of the HGSHS:A (Shor & Orne, 1962), containing five motor challenge items (arm immobility, finger lock, arm rigidity, head shaking inhibition, eye catalepsy). Items are scored dichotomously (0 = fail, 1 = pass), yielding a total score between 0 and 5.

### 2.3. Statistical Analyses

All analyses were conducted in R (version 4.5.1; R Core Team, 2025) using RStudio (version 2025.05.1+513 Mariposa Orchid by Posit Software, 2025). The data were prepared, cleaned, and analyzed in a fully reproducible workflow with *tidyverse* packages (Wickham et al., 2019). Analyses were conducted in several steps corresponding to the psychometric evaluation of the German CIS and its comparison with the HGSHS-5:G. Data were imported from SPSS files originally generated using SPSS version 29 (IBM Corp.) via the *haven* (Wickham et al., 2025) and *here* packages (Müller, 2025) to ensure reproducible file paths.

*Descriptive statistics:* Descriptive statistics (means, standard deviations, skewness, and kurtosis) were computed for all variables with the *sjPlot* (Lüdecke, 2025a), *sjstats* (Lüdecke, 2025b) and *sjlabelled* (Lüdecke, 2022) packages. Missing values were visualized and inspected for patterns using *Amelia* (Honaker et al., 2011). For descriptive tables and summary outputs, *gtsummary* (Sjoberg et al., 2021) was used.

*Item analysis:* Item difficulty, discrimination, and corrected item–total correlations were calculated using *sjPlot* (Lüdecke, 2025a), *sjstats* (Lüdecke, 2025b), *performance* (Lüdecke et al., 2021a) and custom functions from the *strengejacke* package (Lüdecke, 2019). Mean inter-item correlations were inspected as an additional reliability indicator.

*Reliability analysis:* Internal consistency was assessed via Cronbach’s α and McDonald’s ω using the *psych* package (Revelle, 2025). For the CIS, both α and ω total were computed; for the HGSHS-5:G (dichotomous items), α was interpreted as equivalent to KR-20. Confidence intervals for α and item-deleted reliabilities were also examined.

*Exploratory factor analysis (EFA):* Sampling adequacy was tested with the Kaiser–Meyer–Olkin (KMO) statistic and Bartlett’s test of sphericity (Revelle, 2025). To explore the dimensionality of the CIS and HGSHS-5:G, principal components analyses were conducted with *psych* and *parameters* (Lüdecke et al., 2020). Principal component analyses with varimax rotation were run using *parameters*, supplemented by maximum likelihood factor analysis on tetrachoric correlation matrices for the HGSHS-5:G.

*Confirmatory factor analysis (CFA):* Competing one- and two-factor models and a second-order CFA model of the CIS were evaluated using the *lavaan* package (Rosseel, 2012). CFAs were conducted using maximum likelihood estimation (ML), which is appropriate given the approximately symmetric response distributions (skewness: −0.25 to 0.37) and five response categories of the CIS. Model fit was judged according to established criteria (Hu & Bentler, 1999), reporting χ^2^ tests, CFI, TLI, RMSEA with 90% CI, and SRMR. Nested models were compared using χ^2^ difference testing.

*Visualization and reporting:* Factor loadings, item characteristics, and reliability indices were visualized with *see* (Lüdecke et al., 2021b) and *ggplot2* (Wickham, 2016; Wickham et al., 2019). Results were summarized in APA-style tables using *knitr* (Xie, 2014, 2015, 2025) and *report* (Makowski et al., 2023). All analyses were fully reproducible, with package management handled through *pacman* (Rinker & Kurkiewicz, 2018) and selected GitHub packages (strengejacke/strengejacke, easystats/report, easystats/see, easystats/performance).

*Correlation analysis:* Correlations between ordinal CIS items and dichotomous HGSHS-5:G items were computed using mixed polychoric–tetrachoric–polyserial correlations implemented in the *psych::mixedCor()* function (Revelle, 2025). Correlations between the CIS and HGSHS-5:G total scores were computed using Pearson’s r and Spearman’s ρ, as both composite scores can be considered approximately continuous.

### 2.4. Sample Size Considerations

There is ongoing debate about the minimum sample size required for factor-analytic procedures. Rules of thumb typically recommend at least 5–10 participants per item (Gorsuch, 1983), with absolute minimums of n = 100–200 for exploratory factor analysis (MacCallum et al., 1999). For confirmatory factor analysis (CFA), many authors suggest N ≥ 200 as a general lower bound (Kline, 2016), though the exact requirements depend strongly on factor loadings, the number of items per factor, and overall model complexity (Wolf et al., 2013). Reliability analyses such as Cronbach’s α also require sufficient sample sizes for stable estimates, with N ≥ 100 considered adequate for exploratory work, but N ≥ 300 recommended for precise confidence intervals (Bonett, 2002). Against this background, our sample size of approximately 100 participants can be considered sufficient for an initial validation, while acknowledging that larger replications will be required to confirm the CFA results and provide stable estimates of reliability.

## 3. Results

### 3.1. Baseline Characteristics

The analytic sample comprised N = 97 participants allocated to Control (n = 32), PMR (n = 31), and Hypnosis (n = 34). Mean age in the overall sample was 39.22 years (SD = 12.02; range = 20–68); group means were Control: M = 38.09 (SD = 12.13), PMR: M = 39.19 (SD = 12.41), and Hypnosis: M = 40.29 (SD = 11.81). Age was missing for 3 participants overall (Control = 1, PMR = 2, Hypnosis = 0). Overall, 69 (71%) identified as female, 26 (27%) as male, and 2 (2.1%) as diverse (3 missing overall, see Table 1).

For the CIS sum score, the overall mean was M = 17.64 (SD = 7.67; range = 2–35). Group means were Control M = 18.56 (SD = 8.84), PMR M = 17.34 (SD = 7.53), and Hypnosis M = 17.00 (SD = 6.67). Missingness for CIS totaled 6 (Control = 1, PMR = 4, Hypnosis = 1, see Table 1).

For the HGSHS-5:G sum score, the overall mean was M = 2.88 (SD = 1.64; range = 0–5). Group means were Control M = 2.53 (SD = 1.63), PMR M = 3.10 (SD = 1.67), and Hypnosis M = 3.03 (SD = 1.62). Missingness for HGSHS-5:G totaled 6 (Control = 1, PMR = 3, Hypnosis = 2, see Table 1).

### 3.2. Missing Data Analysis

Prior to factor analysis, missing values were inspected across the ten CIS and five HGSHS-5:G items. The proportion of missing data resulted in a total of six incomplete cases (6%). Because this proportion was well below the commonly accepted 10% threshold (Bennett, 2001) and no systematic missingness was observed, listwise deletion was applied, resulting in a valid n = 94 for the factor analyses.

### 3.3. Psychometric Evaluation of the CIS

#### 3.3.1. Sampling Adequacy and Sphericity

The KMO measure of sampling adequacy was 0.81, indicating a “meritorious” level of common variance (Kaiser & Rice, 1974). Individual item measures of sampling adequacy (MSA) all exceeded 0.70 (range 0.75–0.89). Bartlett’s test of sphericity was χ^2^(45) = 353.78, *p* < 0.001, rejecting the null hypothesis of an identity matrix and confirming that the correlation matrix was suitable for factor analysis.

#### 3.3.2. Determining the Number of Factors

To identify the optimal number of underlying dimensions, the *n_factors()* function from the *parameters* package (Lüdecke et al., 2020), which incorporates functions from *psych* (Revelle, 2025) and *psycho* (Makowski, 2018), was applied. This procedure summarizes 19 extraction methods following the Method Agreement Procedure (MAP).

**Figure 1:**
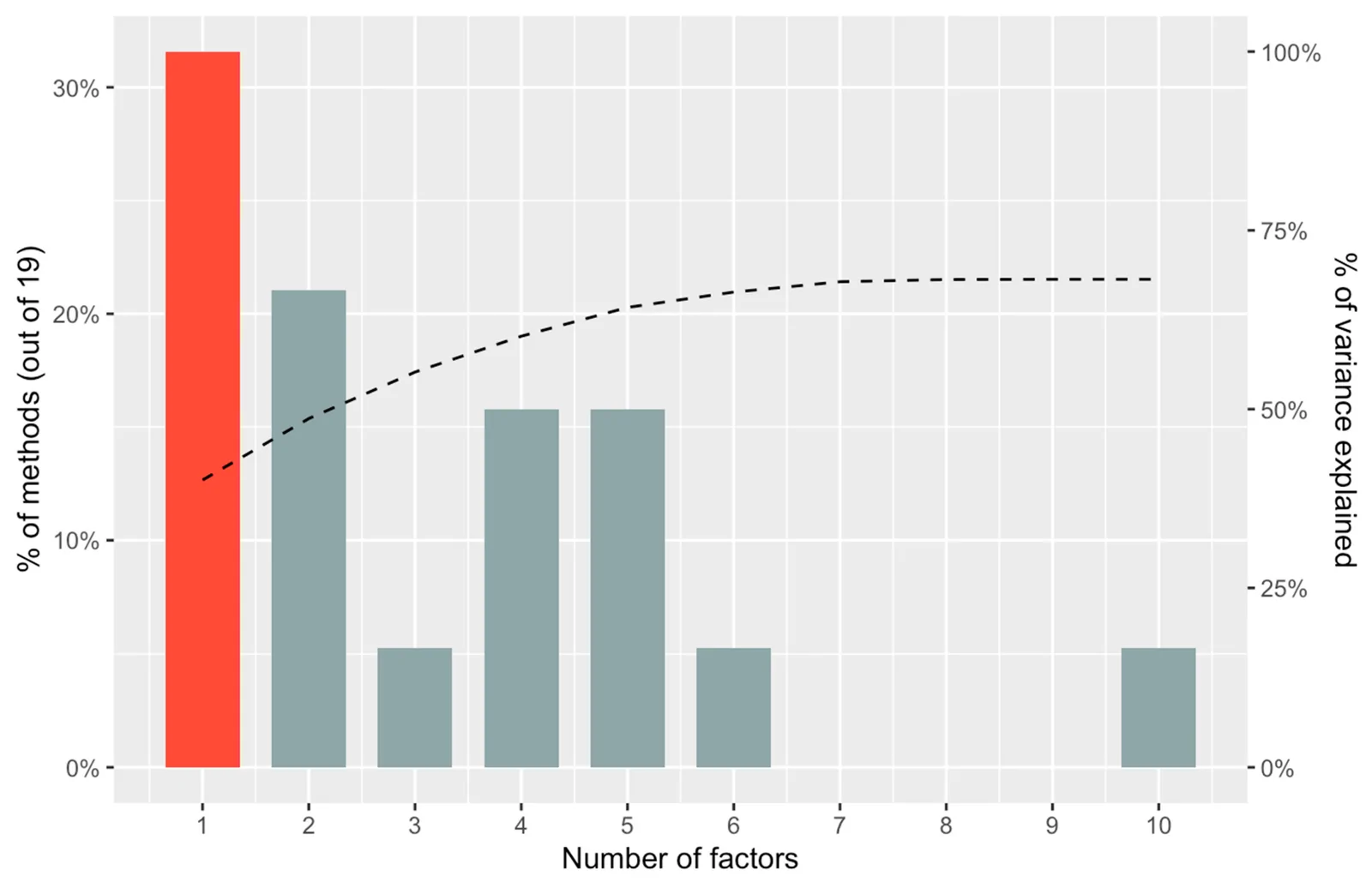
Summary plot showing the result of the Method Agreement Procedure (MAP). Bars showing the percentage of method agreement and the dashed line the predicted percentage of variance explained by factor number.

Recommendations varied across methods: six methods (31.6%) supported a one-factor solution (Acceleration Factor, Scree (SE), Scree (R^2^), VSS Complexity 1, Velicer’s MAP, and BIC), while four (21%) suggested two factors (Optimal Coordinates, Parallel Analysis, Kaiser Criterion, VSS Complexity 2). The first factor accounted for 40% of the total variance, and a two-factor solution explained 48.6%. (see Figure 1 and Table 2). Higher-order suggestions (three to five factors) explained minimal additional variance and were theoretically implausible.

Overall, most indices pointed toward a predominantly unidimensional structure, but several theoretically grounded procedures—particularly the parallel analysis and Kaiser criterion—supported a two-factor solution.

Given this mixed evidence and the theoretical distinction in older studies between a factor Hypnotic Performance and an Absorption/Imagination factor, a two-factor model was retained for further evaluation in the confirmatory analyses.

#### 3.3.3. Component Structure of the CIS

Two principal-component analyses (PCA) were performed using *parameters::principal_components()* with varimax rotation to explore the component structure of the ten CIS items. In the one-component model, all items loaded positively on a single principal component (PC1; loadings = 0.55–0.78; see Table 3). The individual item measures of sampling adequacy (MSA) ranged from 0.75 to 0.89, indicating sampling adequacy at the item level. This single component accounted for 43.14% of the total variance in the item set.

A second PCA specifying two components produced a clearer separation of content domains. After varimax rotation, loadings on PC1 ranged from 0.63 to 0.77 for items 1, 2, 3, 4,7 and 10, representing imaginative absorption and perceptual alteration, whereas loadings on PC2 ranged from 0.60 to 0.82 for items 5, 6, 8 and 9, representing motor or behavioral enactment (see Table 3). Items 5 and 10 showed notable cross-loadings (item complexity = 1.95 and 2.00, respectively), indicating that they share variance across both components.

Together, the two components explained 55.24% of the total variance (PC1 = 30.85%; PC2 = 24.38%). These findings suggest that while the CIS is largely dominated by a general imagination component, a two-component structure may better capture the variance pattern among items.

#### 3.3.4. Hierarchical Reliability Analysis

A hierarchical reliability analysis using *omega()* (Revelle, 2025) was conducted to estimate the proportion of variance attributable to general and group factors within the CIS. The results indicated good internal consistency for the total scale (Cronbach’s α = 0.85; Guttman’s λ_6_ = 0.87). The total omega (ω_t_) was 0.87, suggesting that 87% of the total score variance reflects common variance among items.

**Figure 2:**
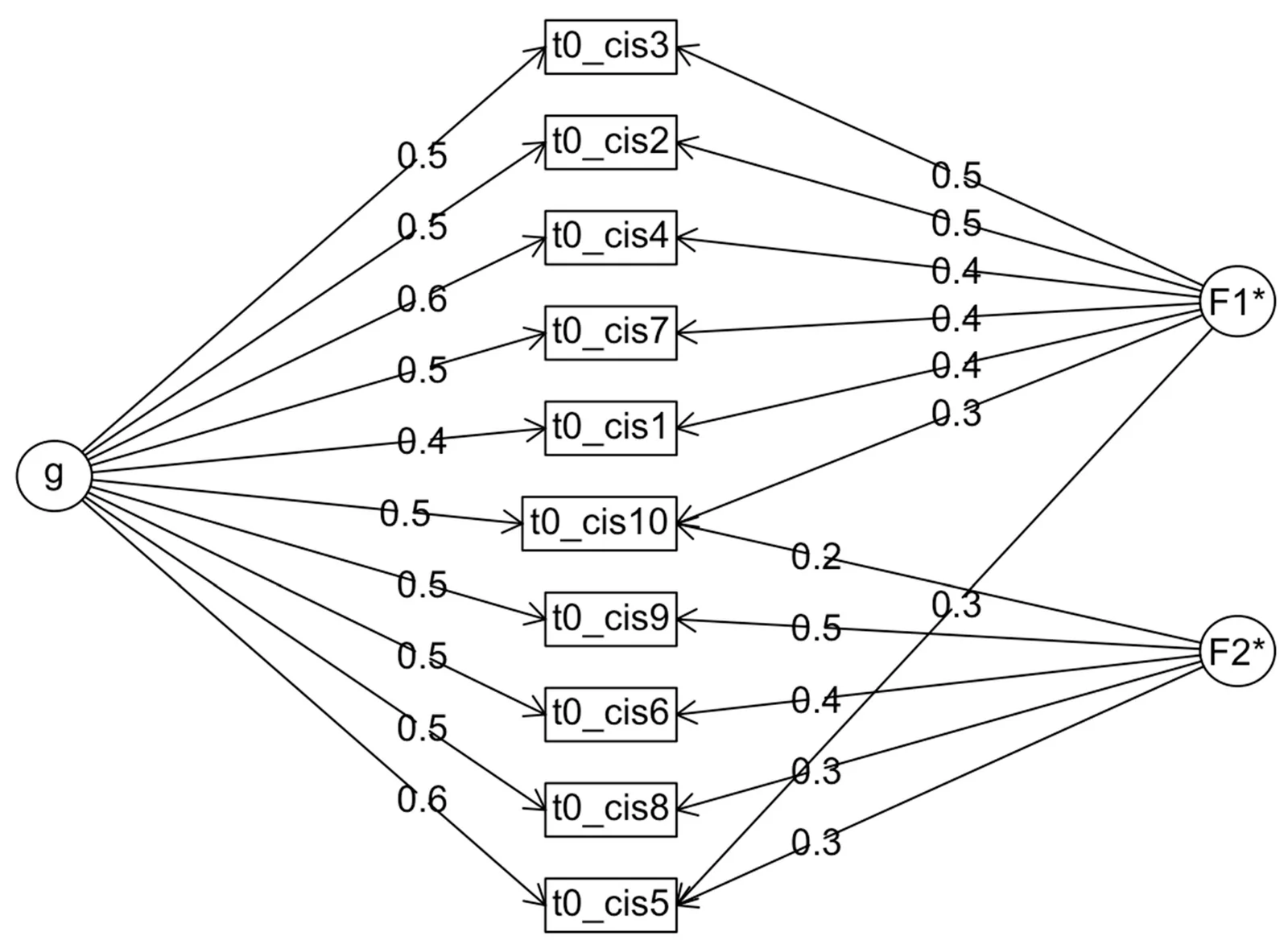
Graphical representation of the one (g) versus the theoretical two-factor solution (F1 and F2) from the omega analysis. * Subscale reliabilities: ω_t_ = 0.81 for F1 and ω_t_ = 0.75 for F2, with g ω_h_ = 0.49 and 0.50.

The hierarchical omega (ω_h_) was 0.62, indicating that approximately 62% of the reliable variance can be attributed to a single general factor of imaginative suggestibility, while the remaining variance reflects group or subfactor influences. The asymptotic ω_h_ value of 0.71 supports the stability of this general factor across samples. The Schmid–Leiman decomposition showed that the general factor explained 60% of the common variance, whereas the two group factors (F1* and F2*, see Table 4) accounted for smaller proportions (sum of squares = 1.10 and 0.67, respectively). Fit indices indicated an acceptable fit for the bifactor model (χ^2^(26) = 62.08, *p* < 0.001; RMSEA = 0.117, 90% CI [0.081, 0.157]; BIC = −57.65). In contrast, a model including only a single general factor showed poorer fit (χ^2^(35) = 107.27, *p* < 0.001; RMSEA = 0.143, 90% CI [0.114, 0.176]; BIC = −53.91). Measures of factor score adequacy showed that factor scores correlated moderately to strongly with their latent factors (r_(_g_)_ = 0.79; r_(_F1_)_ = 0.65; r_(_F2_)_ = 0.62), with corresponding squared multiple correlations (R^2^) of 0.63, 0.43, and 0.38, respectively. The subscale reliabilities indicated ω_t_ = 0.81 for F1* and ω_t_ = 0.75 for F2*, with corresponding ω_h_ = 0.49 and 0.50, respectively (see Table 4 and Figure 2). Overall, these results confirm that the CIS primarily reflects a dominant general factor of imaginative suggestibility, while smaller but meaningful proportions of reliable variance are captured by two subordinate group factors related to Imagination/Absorption and Performance/Perceptual Alteration.

#### 3.3.5. Reliability and Item Analysis

Item-level statistics for the German CIS are presented in Table 5. Across the ten items, the proportion of missing data was consistent at 6%, indicating good completion rates. Item means ranged from 1.60 to 2.10 (SD = 1.07–1.25), showing moderate endorsement across imaginative scenarios on the 0–5 response scale. Skewness values were close to zero (−0.25 ≤ skew ≤ 0.37), suggesting approximately symmetric response distributions. Item difficulty indices (mean item score/maximum possible score) ranged between 0.40 and 0.52, indicating balanced item difficulty. Corrected item–total correlations (discrimination indices) ranged from 0.45 to 0.69, demonstrating that all items contributed meaningfully to the total score. No item deletion would have improved internal consistency, as “α if deleted” values varied narrowly between 0.82 and 0.84. The mean inter-item correlation (MIIC) was 0.36, falling squarely within the recommended 0.15–0.50 range for homogeneous but non-redundant items (Clark & Watson, 1995). The overall Cronbach’s α = 0.85 indicates good internal consistency, comparable to previous validations of the CIS, and confirms that the ten items form a coherent unidimensional scale of imaginative suggestibility.

#### 3.3.6. Confirmatory Factor Analysis (CFA)

To test the potential multidimensionality of the CIS more formally, confirmatory factor analyses were conducted using *lavaan* (Rosseel, 2012). Three competing models were estimated: (1) a unidimensional model representing general imaginative suggestibility, (2) a correlated two-factor model representing Imagination/Absorption and Performance/Perceptual Alteration, and (3) to address the possibility of a hierarchical factor structure, a second-order CFA model was additionally estimated in which the two first-order factors (Imagery/Absorption and Performance/Perceptual Alteration) load onto a superordinate CIS factor.

The one-factor model showed poor-to-marginal fit (see Table 6): χ^2^(35) = 89.95, *p* < 0.001; CFI = 0.820; TLI = 0.769; RMSEA = 0.129 (90% CI [0.097, 0.162]); SRMR = 0.082.

The two-factor model provided a statistically superior fit: χ^2^(34) = 75.98, *p* < 0.001; CFI = 0.863; TLI = 0.818; RMSEA = 0.115 (90% CI [0.080, 0.149]); SRMR = 0.073.

A chi-square difference test confirmed the better fit of the two-factor model, Δχ^2^(1) = 13.97, *p* < 0.001. The two latent factors were highly correlated (r = 0.80, *p* < 0.001).

The second-order model yielded fit indices identical to those of the correlated two-factor model (χ^2^(34) = 75.98, CFI = 0.863, RMSEA = 0.115 [0.080, 0.149], SRMR = 0.073), with a chi-square difference of Δχ^2^(1) = ~0, *p* = 1.0, indicating that the two models are statistically equivalent. This equivalence is a mathematical consequence of specifying exactly two first-order factors: the second-order factor merely reparametrizes the interfactor correlation (r = 0.80) as a higher-order loading without imposing additional constraints. Given this equivalence, the correlated two-factor model is retained as the primary solution on grounds of parsimony and interpretability. Evidence for a general higher-order factor is instead provided by the hierarchical omega and bifactor analyses reported in Section 3.3.4, which demonstrated that a dominant general factor accounts for 60% of common variance (ω_h_ = 0.62).

These results indicate that, while the CIS can be treated as predominantly unidimensional for practical purposes, its structure may be best represented by two correlated factors, reflecting both imaginative absorption and performance-related perceptual alteration.

### 3.4. Psychometric Results of the HGSHS-5:G

The HGSHS-5:G demonstrated satisfactory psychometric properties. Bartlett’s test confirmed sufficient inter-item correlations (χ^2^(10) = 79.24, *p* < 0.001), and the KMO measure indicated middling sampling adequacy (0.74). Factor analyses consistently supported a unidimensional structure. Internal consistency was acceptable for a short dichotomous scale (Cronbach’s α = 0.68; mean inter-item correlation = 0.30). Detailed results of the factor analysis and item-level statistics are provided in Supplementary Materials.

### 3.5. Comparison Between the CIS and HGSHS-5:G

To examine the relationship between imaginative suggestibility (CIS) and behavioral hypnotic responsiveness (HGSHS-5:G), both item-level and scale-level correlations were computed. Mixed correlations (polychoric for ordinal CIS items and tetrachoric for dichotomous HGSHS-5:G items) revealed a predominantly positive pattern of associations between corresponding items of both instruments, with coefficients ranging from r = 0.18 to 0.56 (see Table 7). Stronger cross-measure correlations were observed for the CIS items involving perceptual or motor imagery (CIS Items 3–5, 7, 10) and the HGSHS-5:G items reflecting behavioral enactment (HGSHS-5:G Items 3–5). At the aggregate level, the total CIS score correlated moderately with the HGSHS-5:G total score, r = 0.52, *p* < 0.001.

This finding indicates that individuals scoring high on imaginative and absorptive suggestibility also tend to exhibit stronger behavioral responses to standardized hypnotic suggestions. The moderate magnitude of this association supports convergent validity between the two measures while demonstrating that they assess related but non-redundant constructs—cognitive–imaginative versus behavioral aspects of hypnotic suggestibility. Correlations among individual CIS items and the HGSHS-5:G sum score ranged from r = 0.20 to 0.42 (*p* < 0.05), further reinforcing this pattern.

## 4. Discussion

The present study represents the first validation of the German version of the CIS in a clinical sample of individuals with mild to moderate depressive symptoms. The study further compared the German CIS with the HGSHS-5:G to examine convergent and discriminant aspects of imaginative versus behavioral suggestibility. Establishing a validated German CIS was necessary, as no standardized measure of imaginative suggestibility currently exists for German-speaking clinical populations.

Overall, the results demonstrate that the German CIS shows good internal consistency and preliminary but limited evidence for a two-factor structure—though absolute CFA fit was suboptimal in the present sample, which should be interpreted in light of the relatively small sample size—alongside a moderate positive correlation with the HGSHS-5:G, supporting convergent validity while indicating that imaginative and behavioral suggestibility represent related but distinct constructs.

### 4.1. Psychometric Properties of the CIS

The German CIS demonstrated good internal consistency, comparable to or exceeding reliability estimates reported in earlier English-language studies of the CIS in experimental and clinical samples (Wilson & Barber, 1978; Sheehan et al., 1978; Laidlaw & Large, 1997). Exploratory and confirmatory factor analyses indicated that a two-factor model provided a slightly better fit compared to a unidimensional structure. The factors, interpretable as Imagination/Absorption and Performance/Perceptual Alteration, are consistent with previous factor-analytic work suggesting that imaginative suggestibility encompasses multiple dimensions (Hilgard et al., 1981; Laurence et al., 2008). This is tentatively consistent with the view that the CIS may capture multiple dimensions of imaginative suggestibility, though the high interfactor correlation (r = 0.80) and suboptimal model fit in the present sample limit the strength of this conclusion.

Overall, these findings are broadly consistent with earlier psychometric and comparative work on the CIS, including studies reporting acceptable reliability as well as investigations of its factor structure and relationship to other suggestibility measures (e.g., Wilson & Barber, 1978; Hilgard et al., 1981; McConkey et al., 1979). The present study extends this work by providing initial validation data for a German clinical sample with depressive symptoms. The CFA results extend the EFA findings by providing preliminary support for a correlated two-factor solution as a better representation of the CIS structure than the unidimensional model. Neither model reached conventional thresholds for acceptable fit (CFI > 0.90, RMSEA < 0.08), which should be interpreted in light of the relatively small sample size. The two latent dimensions were highly correlated (r = 0.80), indicating substantial shared variance; they may therefore be better understood as correlated facets of a broader imaginative suggestibility construct than as independent dimensions. This is consistent with prior work on multidimensional structures of suggestibility scales (McConkey et al., 1980; Piesbergen & Peter, 2006; Polito et al., 2014; Zahedi et al., 2024) and suggests that a two-dimensional conceptualization warrants further investigation, though firm conclusions about subscale utility are premature pending replication in larger samples.

### 4.2. Psychometric Properties of the HGSHS-5:G

The HGSHS-5:G was confirmed as a reliable and unidimensional instrument, consistent with recent German validation studies (Riegel et al., 2021; Zech et al., 2024). Although internal consistency was only modest (α = 0.68), this level of reliability is acceptable given the brevity and heterogeneity of the five dichotomous motor challenge items. This supports the scale’s use as an efficient behavioral index of hypnotic responsiveness, even in clinical populations with mild depressive symptoms.

### 4.3. Convergent Validity and Distinctiveness

The CIS and the HGSHS-5:G showed a moderate positive correlation (r = 0.52), with a disattenuated correlation of approximately 0.68, indicating substantial shared variance alongside clear conceptual distinctiveness. This result is broadly consistent with earlier English-language studies comparing the CIS with the HGSHS:A. For example, McConkey et al. (1979) reported a modest positive correlation between CIS and HGSHS:A scores (r = 0.28), while demonstrating that the two instruments reflect largely independent underlying dimensions. Importantly, the HGSHS:A comprises a broader range of hypnotic suggestions, whereas the HGSHS-5:G focuses exclusively on motor challenge items. Differences in scale composition, sample characteristics, and clinical context may therefore partly account for the stronger correlation observed in the present study. Overall, these findings support the view that imaginative suggestibility, as assessed by the CIS, and behavioral hypnotic responsiveness under induction, as assessed by the HGSHS-5:G, are related but non-redundant constructs.

### 4.4. Clinical and Theoretical Implications

From a clinical perspective, these results are highly relevant. Hypnotic suggestibility has been linked to treatment outcomes in hypnotherapy (Montgomery et al., 2011). In addition, hypnotherapy has been shown to be non-inferior to cognitive behavioral therapy in the treatment of mild to moderate depressive episodes (Fuhr et al., 2021). The CIS may be particularly valuable as a brief, non-induction-based screening tool for imaginative abilities in patients with depression, where imagery-based interventions (e.g., guided imagery, hypnosis, or mental imagery training) have shown therapeutic potential (Holmes et al., 2016; Renner et al., 2017). For clinical use, the total CIS score currently represents the more robust index, as the preliminary nature of the two-factor solution and the high interfactor correlation (r = 0.80) warrant caution in interpreting subscale scores until replication in larger samples is available. The moderate convergence between CIS and HGSHS-5:G also indicates that both instruments tap into core mechanisms of suggestibility, but in complementary ways: one through imaginative absorption, the other through motor challenge performance. This suggests that the two measures may be differentially sensitive to individual differences in cognitive style and clinical symptomatology.

### 4.5. Limitations

A key limitation of the present study concerns the relatively modest sample size (N ≈ 94), which falls below many recommended thresholds for confirmatory factor analysis and reliability estimation (Kline, 2016; Wolf et al., 2013). For exploratory factor analysis specifically, the adequacy of a given sample size depends critically on the level of communality and factor overdetermination: while 100–200 cases may suffice when communalities are high (>0.60) and factors are well-defined, lower communalities and weakly determined factors increase the required sample to 300 or more (MacCallum et al., 1999). In the present data, item communalities ranged from 0.43 to 0.67 (M = 0.55), suggesting that the EFA results in particular should be interpreted with appropriate caution. Although our sample size was sufficient to support exploratory analyses, the structural findings remain preliminary. Future research with larger and more diverse samples is needed to confirm the robustness of the two-factor structure of the CIS and to refine its psychometric properties. Moreover, because the EFA and the CFA were performed on the same dataset and the CIS comprises a small and heterogeneous set of items, the structural validation results should be interpreted with caution, as this design limits the robustness of the factor-analytic evidence.

### 4.6. Strengths

Despite these limitations, the present study has several notable strengths. It represents the first validation of the German CIS in a clinical sample of individuals with mild to moderate depressive symptoms, addressing a critical gap in suggestibility assessment for German-speaking populations. The study employed a rigorous psychometric approach, combining exploratory and confirmatory factor analyses and examining both internal consistency and convergent validity with the HGSHS-5:G. By integrating a non-induction-based measure of imaginative suggestibility (CIS) and a behavioral measure of hypnotic performance (HGSHS-5:G), the study provides a comprehensive and differentiated assessment of suggestibility, highlighting the multidimensional nature of the construct.

## 5. Conclusions

This study represents the first systematic attempt to evaluate a German version of the CIS, filling a critical gap in the literature. As no prior German-language validation has been available, the present findings provide an important first benchmark for the use of the CIS in clinical and research contexts. Conducted in a sample of individuals with mild to moderate depressive symptoms, the study extends previous work by demonstrating that the German CIS is a reliable instrument for assessing imaginative suggestibility in clinical populations, with preliminary evidence for a two-factor structure pending replication in larger independent samples. While replication with larger and more diverse samples is essential, its brief, non-induction-based format may facilitate the assessment of imaginative abilities in both clinical and experimental settings, complementing existing behavioral measures of suggestibility.

## Figures and Tables

**Table 1:** Table summarizing patients’ baseline characteristics, VAS, CIS and HGSHS-5:G scores.

			Treatment Received	
Variable	Overall N = 97 ^1^	Control N = 32 ^1^	PMR N = 31 ^1^	Hypnosis N = 34 ^1^
Age	39.22 (12.02, 20.00, 68.00)	38.09 (12.13, 22.00, 68.00)	39.19 (12.41, 20.00, 64.00)	40.29 (11.81, 20.00, 60.00)
(Missing)	3	1	2	0
Sex				
Woman	69/97 (71%)	27/32 (84%)	22/31 (71%)	20/34 (59%)
Man	26/97 (27%)	5/32 (16%)	8/31 (26%)	13/34 (38%)
Diverse	2/97 (2.1%)	0/32 (0%)	1/31 (3.2%)	1/34 (2.9%)
(Missing)	3	1	2	0
VAS	70.56 (11.58, 42.00, 100.00)	69.19 (11.84, 42.00, 100.00)	70.16 (11.92, 45.00, 99.00)	72.24 (11.13, 42.00, 95.00)
(Missing)	2	1	1	0
CIS score	17.64 (7.67, 2.00, 35.00)	18.56 (8.84, 2.00, 35.00)	17.34 (7.53, 3.00, 31.00)	17.00 (6.67, 2.00, 33.00)
(Missing)	6	1	4	1
HGSHS-5:G score	2.88 (1.64, 0.00, 5.00)	2.53 (1.63, 0.00, 5.00)	3.10 (1.67, 0.00, 5.00)	3.03 (1.62, 0.00, 5.00)
(Missing)	6	1	3	2

^1^ Mean (SD, Min, Max); n/N (%); VAS = Visual Analog Scale (stress); HGSHS = Harvard Group Scale of Hypnotic Susceptibility; CIS = Creative Imagination Scale.

**Table 2:** Summary of factor-extraction recommendations from the Method Agreement Procedure (MAP).

N Factors ^1^	Method	Family	Cumulative Variance
1	Acceleration factor	Scree	0.400
1	Scree (SE)	Scree_SE	0.400
1	Scree (R^2^)	Scree_SE	0.400
1	VSS complexity 1	VSS	0.400
1	Velicer’s MAP	Velicers_MAP	0.400
1	BIC	BIC	0.400
2	Optimal coordinates	Scree	0.486
2	Parallel analysis	Scree	0.486
2	Kaiser criterion	Scree	0.486
2	VSS complexity 2	VSS	0.486
3	CNG	CNG	0.551
4	beta	Multiple_regression	0.601
4	t	Multiple_regression	0.601
4	*p*	Multiple_regression	0.601

^1^ Number of suggested factors by method used.

**Table 3:** Overview of the component structure from principal components analysis for one- and two-component solutions of the CIS.

Principal Component Analysis with One Component					Principal Component Analysis with Two Components				
Variable	PC1	Complexity	Uniqueness	MSA	PC1	PC2	Complexity	Uniqueness	MSA
t0_cis1	0.6	1	0.64	0.8	0.63	0.17	1.14	0.57	0.8
t0_cis2	0.71	1	0.49	0.83	0.77	0.18	1.11	0.37	0.83
t0_cis3	0.67	1	0.55	0.8	0.76	0.12	1.05	0.4	0.8
t0_cis4	0.72	1	0.48	0.79	0.67	0.31	1.42	0.45	0.79
t0_cis5	0.78	1	0.4	0.84	0.51	0.6	1.95	0.37	0.84
t0_cis6	0.61	1	0.63	0.79	0.17	0.76	1.1	0.39	0.79
t0_cis7	0.62	1	0.61	0.75	0.68	0.15	1.09	0.51	0.75
t0_cis8	0.61	1	0.63	0.89	0.26	0.65	1.32	0.52	0.89
t0_cis9	0.55	1	0.7	0.79	0.04	0.82	1.01	0.33	0.79
t0_cis10	0.67	1	0.55	0.82	0.48	0.47	2	0.55	0.82
The single principal component (varimax rotation) accounted for 43.14% of the total variance of the original data.					The 2 principal components (varimax rotation) accounted for 55.24% of the total variance of the original data (PC1 = 30.85%, PC2 = 24.38%).				

**Table 4:** Table summarizing factor scores for one general factor g and two theoretical two-factor solutions, F1 and F2, from *omega* analysis.

Variable	English	German	g	F1*	F2*
t0_cis1	Dictionaries Hand	Wörterbücher Hand	0.43	0.36	
t0_cis2	Water jet Hand	Wasserstrahl Hand	0.54	0.49	
t0_cis3	Narcotics Hand	Betäubungsmittel Hand	0.49	0.5	
t0_cis4	Drinking cool mountain water	Kühles Bergwasser trinken	0.55	0.41	
t0_cis5	Smelling oranges	Orange riechen	0.65	0.27	0.3
t0_cis6	Beautiful music	Wunderschöne Musik	0.52		0.42
t0_cis7	Sun on your hand	Sonne auf die Hand	0.45	0.4	
t0_cis8	Time slows down	Zeit verlangsamt	0.49		0.31
t0_cis9	Thinking back to elementary school	Zeit zurückzudenken Grundschule	0.49		0.5
t0_cis10	Sun on the beach	Sonne am Strand	0.52	0.26	0.2

* Subscale reliabilities: ωt = 0.81 for F1 and ωt = 0.75 for F2, with g ωh = 0.49 and 0.50.

**Table 5:** Item-level descriptive and reliability statistics for the CIS with one factor.

Row	Missings	Mean	SD	Skew	Item Difficulty	Item Discrimination	α If Deleted
cis1	6.00%	2.06	1.18	−0.25	0.52	0.49	0.84
cis2	6.00%	1.81	1.20	0.19	0.45	0.61	0.83
cis3	6.00%	1.60	1.18	0.31	0.40	0.56	0.84
cis4	6.00%	1.61	1.08	0.37	0.40	0.61	0.83
cis5	6.00%	1.78	1.19	0.10	0.44	0.69	0.82
cis6	6.00%	1.60	1.25	0.24	0.40	0.52	0.84
cis7	6.00%	1.64	1.13	0.08	0.41	0.52	0.84
cis8	6.00%	1.66	1.24	0.19	0.41	0.52	0.84
cis9	6.00%	1.80	1.22	0.18	0.45	0.45	0.84
cis10	6.00%	2.10	1.07	−0.14	0.52	0.57	0.83

Mean inter-item-correlation = 0.364. Cronbach’s α = 0.850.

**Table 6:** Table summarizing fit indices for the one- and two-factor CFA models of the CIS.

Model	χ^2^ (df)	*p*	CFI	TLI	RMSEA [90% CI]	SRMR	Δχ^2^ (df Diff)	Factor Correlation
CIS 1-Factor CFA	89.95 (35)	<0.001	0.820	0.769	0.129 [0.097, 0.162]	0.082	-	-
CIS 2-Factor CFA	75.98 (34)	<0.001	0.863	0.818	0.115 [0.080, 0.149]	0.073	13.97 (1), *p* < 0.001	r = 0.80
CIS 2nd-Order CFA ^†^	75.98 (34)	<0.001	0.863	0.818	0.115 [0.080, 0.149]	0.073	~0 (1), *p* = 1.0	-

^†^ The second-order CFA model specifies Imagery and Performance as first-order factors loading onto a superordinate CIS factor. With exactly two first-order factors, the model is not identified without constraints; a residual variance constraint was therefore applied (Imagery ~~ 0*Imagery). This renders the model statistically equivalent to the correlated two-factor solution (Δχ^2^(1) = ~0, *p* = 1.0), confirming that the interfactor correlation (r = 0.80) is fully accounted for by the higher-order factor. The bifactor analysis in Section 3.3.4 provides a more informative test of the higher-order structure.

**Table 7:** Mixed correlations (polychoric/tetrachoric) between all CIS and all HGSHS-5:G items.

	*t0_cis1*	*t0_cis2*	*t0_cis3*	*t0_cis4*	*t0_cis5*	*t0_cis6*	*t0_cis7*	*t0_cis8*	*t0_cis9*	*t0_cis10*	*t0_hgshs_1*	*t0_hgshs_2*	*t0_hgshs_3*	*t0_hgshs_4*	*t0_hgshs_5*
*t0_cis1*															
*t0_cis2*	0.59														
*t0_cis3*	0.30	0.45													
*t0_cis4*	0.37	0.47	0.54												
*t0_cis5*	0.40	0.47	0.39	0.63											
*t0_cis6*	0.24	0.27	0.28	0.26	0.51										
*t0_cis7*	0.28	0.43	0.53	0.29	0.32	0.34									
*t0_cis8*	0.31	0.26	0.33	0.32	0.41	0.44	0.29								
*t0_cis9*	0.21	0.27	0.15	0.28	0.41	0.45	0.13	0.37							
*t0_cis10*	0.23	0.40	0.36	0.42	0.45	0.30	0.48	0.33	0.42						
*t0_hgshs_1*	0.30	0.22	0.35	0.27	0.22	0.18	0.25	0.29	0.20	0.27					
*t0_hgshs_2*	0.16	0.03	0.00	0.07	−0.08	0.08	0.02	0.12	0.02	0.10	0.05				
*t0_hgshs_3*	0.18	0.16	0.37	0.40	0.33	0.14	0.23	0.14	0.12	0.27	0.44	0.33			
*t0_hgshs_4*	0.33	0.34	0.27	0.56	0.32	0.17	0.23	0.21	0.19	0.18	0.43	0.36	0.55		
*t0_hgshs_5*	0.28	0.27	0.39	0.39	0.20	0.24	0.29	0.20	0.30	0.28	0.60	0.37	0.70	0.63	

Note. Values below the diagonal represent mixed polychoric (CIS × CIS), tetrachoric (HGSHS-5:G × HGSHS-5:G), and polychoric–tetrachoric (CIS × HGSHS-5:G) correlations. Diagonal entries (·) are omitted. CIS = Creative Imagination Scale; HGSHS-5:G = Harvard Group Scale of Hypnotic Susceptibility, Short Form, German version.

## Data Availability

The datasets generated and/or analyzed during the current study are available from the corresponding author upon reasonable request for scientific purposes and in compliance with data protection regulations.

## Supplementary Materials

The following supporting information can be downloaded at: https://www.mdpi.com/article/10.3390/bs16091502/s1, Figure S1: Determining the number of factors using the Method Agreement Procedure (MAP) for HGSHS-5:G. Bars showing the percentage of method agreement and the dotted line the prognosted percentage of variance explained by factor number; Table S1: HGSHS-5:G Rotated loadings from Principal Components Analysis (varimax-rotation); Table S2: Item level descriptive statistics, difficulty, discrimination and Cronbach’s alpha.

## Abbreviations

The following abbreviations are used in this manuscript:

CIS	Creative Imagination Scale
HGSHS:A	Harvard Group Scale of Hypnotic Susceptibility, Form A
HGSHS-5:G	Harvard Group Scale of Hypnotic Susceptibility, 5-item German short form
CFA	Confirmatory Factor Analysis
EFA	Exploratory Factor Analysis
α	Cronbach’s Alpha
ω	McDonald’s Omega
KMO	Kaiser–Meyer–Olkin
MSA	Measure of sampling adequacy
RMSEA	Root Mean Square Error of Approximation
TLI	Tucker–Lewis-Index
CFI	Comparative Fit Index
SRMR	Standardized Root Mean Square Residual
CI	Confidence Interval
DRKS	Deutsches Register Klinischer Studien (German Clinical Trials Register)
