# Supplementary material for: Validation of the German Version of the Creative Imagination Scale (CIS) in Individuals with Mild to Moderate Depressive Symptoms: A Comparison with the HGSHS-5:G
Source: Behav Sci (Basel). 2026 Aug 27;16(9):1502. doi: 10.3390/bs16091502 (PMC13603292; doi:10.3390/bs16091502)
Supplement: Supplementary file 1 [file behavsci-16-01502-s001.zip › behavsci-4168939-supplementary.pdf]

behavioral sciences

MDPI

Validation of the German Version of the Creative Imagination Scale (CIS) in Individuals with Mild to Moderate Depressive Symptoms: A Comparison with the HGSHS-5:G

SUPPLEMENTAL MATERIAL S1

S1. Psychometric evaluation of the HGSHS-5:G

S1.1. Sampling adequacy and sphericity

Preliminary diagnostics confirmed that the HGSHS-5:G data were appropriate for factor analysis. Bartlett's test of sphericity was significant, $\chi^2(10) = 79.24$, $p < .001$, indicating sufficient inter-item correlations. The overall Kaiser–Meyer–Olkin (KMO) measure of sampling adequacy was .74, reflecting a "middling" level of shared variance (Kaiser, 1974). Individual KMO values ranged between .70 and .79 (Item 1 = .74; Item 2 = .73; Item 3 = .75; Item 4 = .79; Item 5 = .70), confirming acceptable factorability for all five binary items.

S1.2. Determining the number of factors

The Method Agreement Procedure (MAP) implemented through the *parameters* package (Lüdecke et al., 2020; Makowski, 2018) evaluated 13 extraction methods (see Figure S1). A one-factor solution was supported by 9 of 13 methods (69 %), including the Bartlett, Anderson, Optimal-Coordinates, Acceleration-Factor, Parallel-Analysis, Kaiser-Criterion, Scree (SE), Scree (R^2), and Velicer's MAP procedures. Cumulative variance explained increased modestly from 34.3 % for one factor to 40.4 % for two factors and 42.4 % for three, indicating that additional factors added minimal explanatory power. Together, these findings consistently favored a unidimensional structure for the HGSHS-5:G.

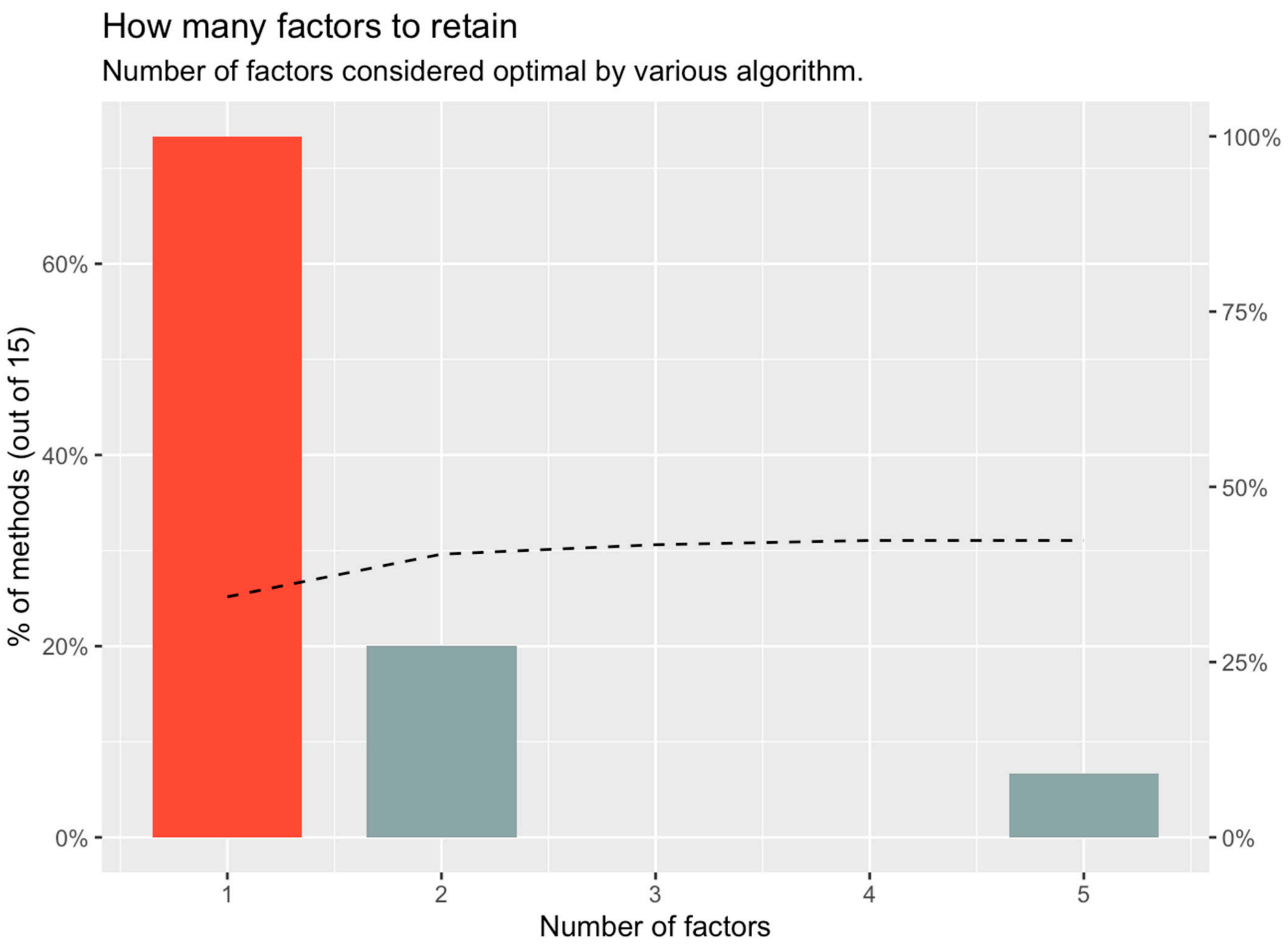

Figure S1. Determining the number of factors using the Method Agreement Procedure (MAP) for HGSHS-5:G. Bars showing the percentage of method agreement and the dotted line the prognosted percentage of variance explained by factor number.

S1.3. Principal components analysis

A principal-components analysis (PCA) with varimax rotation further confirmed a strong general component. All five items loaded positively on a single component with loadings ranging from .43 to .81 (see Table S1). The unique principal component accounted for 44.95 % of the total variance in the item set. Individual item complexities were all = 1.00, and uniqueness values ranged from .34 to .82, indicating moderate to high communalities. The results suggest that the five behavioral tasks measure a single latent dimension of hypnotic responsiveness.

Table S1. HGSHS-5:G Rotated loadings from Principal Components Analysis (varimax-rotation)

Variable	PC1	Complexity	Uniqueness	MSA
t0_hgshs_1	0.60	1	0.64	0.74
t0_hgshs_2	0.43	1	0.82	0.73
t0_hgshs_3	0.74	1	0.45	0.75
t0_hgshs_4	0.71	1	0.50	0.79
t0_hgshs_5	0.81	1	0.34	0.70

S1.4. Reliability and item analysis

Item-level analyses showed balanced item difficulty and acceptable discrimination indices (Table S2). The proportion of missing responses was 6 % for each item. Mean item scores ranged from 0.51 to 0.62 (on the 0–1 scale), indicating moderate endorsement. Item discrimination coefficients ranged from .24 to .60, and "α if deleted" values remained between .55 and .71, demonstrating that no single item degraded reliability. The mean inter-item correlation was .30, within the recommended range of .15–.50 (Clark & Watson, 1995). Overall internal consistency was satisfactory given the short length and dichotomous format of the scale, Cronbach's α = .68.

Table S2. Item level descriptive statistics, difficulty, discrimination and Cronbach´s alpha.

Row	Missings	Mean	SD	Skew	Item Difficulty	Item Discrimination	α if deleted
HGSHS 1	6.00 %	0.62	0.49	-0.49	0.62	0.35	0.66
HGSHS 2	6.00 %	0.57	0.50	-0.31	0.57	0.24	0.71
HGSHS 3	6.00 %	0.60	0.49	-0.40	0.60	0.51	0.59
HGSHS 4	6.00 %	0.51	0.50	-0.04	0.51	0.48	0.60
HGSHS 5	6.00 %	0.59	0.50	-0.35	0.59	0.60	0.55

Mean inter-item-correlation=0.297 · Cronbach's α=0.679

Across all procedures, the HGSHS-5:G demonstrated adequate reliability and a clear one-factor structure. The findings replicate previous validations of the short form, confirming that the five behavioral items collectively capture a single dimension of hypnotic responsiveness. This unidimensional structure justifies the use of the total HGSHS-5:G score as a global measure of hypnotic susceptibility in subsequent analyses.
